# Effectiveness of rehabilitation for working-age patients after a total hip arthroplasty: a comparison of usual care between the Netherlands and Germany

**DOI:** 10.1186/s12891-023-06654-w

**Published:** 2023-06-27

**Authors:** Annet Wijnen, Gesine H. Seeber, Günter Dietz, Baukje Dijkstra, Johan S. Dekker, Karin M. Vermeulen, Geranda E. C. Slager, Aike Hessel, Djordje Lazovic, Sjoerd K. Bulstra, Martin Stevens

**Affiliations:** 1 Department of Orthopedics, University of Groningen, University Medical Center Groningen, Groningen, The Netherlands; 2 University Hospital for Orthopaedics and Trauma Surgery Pius-Hospital, Medical Campus University of Oldenburg, Oldenburg, Germany; 3 Clinic for Orthopedic and Rheumatological Rehabilitation, Reha-Zentrum Am Meer Bad Zwischenahn, Bad Zwischenahn, Germany; 4 Praxis Für Orthopädie, Bad Zwischenahn, Germany; 5 Department of Orthopedics, Medical Center Leeuwarden, Leeuwarden, The Netherlands; 6 Department of Orthopedics, Ommelander Ziekenhuis Groningen, Scheemda, The Netherlands; 7 Department of Epidemiology, University of Groningen, University Medical Center Groningen, Groningen, The Netherlands; 8 Department of Physical Therapy, School of Health Care Studies, Hanze University of Applied Sciences, Groningen, The Netherlands; 9 Deutsche Rentenversicherung Oldenburg, Bremen, Germany

**Keywords:** Orthopedics, Total hip replacement, Rehabilitation, Osteoarthritis, Physiotherapy, Patient satisfaction, Economic evaluation, Cost comparison

## Abstract

**Background:**

Postoperative rehabilitation after primary total hip arthroplasty (p-THA) differs between the Netherlands and Germany. Aim is to compare clinical effectiveness and to get a first impression of cost effectiveness of Dutch versus German usual care after p-THA.

**Methods:**

A transnational prospective controlled observational trial. Clinical effectiveness was assessed with self-reported questionnaires and functional tests. Measurements were taken preoperatively and 4 weeks, 12 weeks, and 6 months postoperatively. For cost effectiveness, long-term economic aspects were assessed from a societal perspective.

**Results:**

124 working-age patients finished the measurements. German usual care leads to a significantly larger proportion (65.6% versus 47.5%) of satisfied patients 12 weeks postoperatively and significantly better self-reported function and Five Times Sit-to-Stand Test (FTSST) results. German usual care is generally 45% more expensive than Dutch usual care, and 20% more expensive for working-age patients. A scenario analysis assumed that German patients work the same number of hours as the Dutch, and that productivity costs are the same. This analysis revealed German care is still more expensive but the difference decreased to 8%.

**Conclusions:**

German rehabilitation is clinically advantageous yet more expensive, although comparisons are less straightforward as the socioeconomic context differs between the two countries.

**Trial registration:**

The study is registered in the German Registry of Clinical Trials (DRKS00011345, 18/11/2016).

**Supplementary Information:**

The online version contains supplementary material available at 10.1186/s12891-023-06654-w.

## Background

Osteoarthritis (OA) is an age-related chronic progressive joint disorder [1]. It is recognized as a substantial source of disability and work absenteeism. Besides significant social and financial costs due to the surgical and medical interventions, it comes along with reduced work ability [2]. For end-stage hip OA, the surgical management of total hip arthroplasty (THA) is mostly indicated [3]. Germany and the Netherlands, with 309 and 238 THAs per 100,000 population, respectively, occupied top positions worldwide in 2017 [4]. In 2018, 31,599 primary total hip arthroplasties were performed in the Netherlands and 239,204 in Germany [5, 6]. With an aging population and increasing numbers of overweight and obese people, hip OA incidence in the Western world will rise [7]. There is an upward trend of OA among the younger working-age population. Within just one decade, the number of patients with hip OA aged 25–60 in Germany increased by about 25% [8], so 63,350 patients under age 65 underwent a primary total hip arthroplasty (p-THA) in 2018 [5]. This working-age population is becoming an increasingly important group, as those patients are expected to return to work after surgery. With higher retirement ages in Western societies, this patient group faces quite a few more years on the job with an artificial joint in place. It is therefore particularly important for them to have an optimal recovery.

THA-related procedures and recovery differ significantly between Germany and the Netherlands. In the Netherlands, as in many other Western countries, there is an increasing tendency to perform p-THA following a fast-track approach. This allows people to leave the hospital within a few days of surgery. A disadvantage is that Dutch patients are minimally supported in their rehabilitation process during hospital admission and after discharge. Postoperative physiotherapy is essentially not covered by Dutch basic health insurance [9]. Patients who need or want postoperative physiotherapy need supplementary insurance or have to pay for it themselves. In practice this leads to a large variation in postoperative rehabilitation, from patients receiving extensive physiotherapy and others receiving no physiotherapy at all. Being minimally supported in the rehabilitation process can lead to suboptimal recovery.

By contrast, in Germany, most patients stay hospitalized for approximately 8–10 days following p-THA [10]. Directly after discharge from the hospital, a 3-week rehabilitation period is followed at a specialized rehabilitation center. The rehabilitation is aimed to reduce OA-related symptoms such as chronic pain and dysfunction and to preserve/restore quality-of-life. Specifically, for the younger working-age population, rehabilitation focuses on reintegration into family and daily as well as working life. During this rehabilitation period, a multi-professional treatment approach is implemented, including but not limited to joint stabilization, muscle strengthening, joint protection training, provision of aids, learning coping strategies for daily life with disabilities, and nutritional counseling [11–13]. After the 3-week rehabilitation period at the rehabilitation center, working-age patients may be offered to enroll in one of the intensified aftercare programs (IRENA/T-RENA) offered by the German pension insurance (DRV). Moreover, according to the remedy’s guidelines (*Heilmittel-Richtlinie*), THA patients fall under specific prescription regulations during the first six months after surgery. Thus, they can receive additional physical therapy, where no limit is set to the amount of physical therapy paid by the statutory health insurance (*gesetzliche Krankenversicherung*). However, it depends on the patient’s health status and the general practitioner or registered orthopedic surgeons’ appreciation of whether additional physiotherapy is prescribed.

Due to the expected increase in the number of hip OA patients in both Germany and the Netherlands, the question arises as to which country’s postoperative p-THA approach is more effective in terms of functional outcome, patient satisfaction, and cost effectiveness. As patients with OA are among the main users of the healthcare system, the expected increase in p-THA will result in a higher socioeconomic burden of OA, especially among employable patients [14]. Insight into the clinical and the cost effectiveness of both postoperative approaches is therefore of the utmost relevance. This study’s primary objective was to compare the clinical effectiveness of the German approach to medical rehabilitation after p-THA with the Dutch approach, where the focus of the analysis was set on working-age patients. It was hypothesized that more intensive medical rehabilitation after p-THA, as performed in Germany, would lead to higher patient satisfaction and better hip function. The secondary objective was to gain a first impression of the rehabilitation costs. Here it was assumed that the German approach, despite an initially higher expenditure burden, would be more cost-effective in the medium term than the Dutch approach.

## Methods

### Study design

The study was conducted as a transnational prospective controlled observational trial analyzing the clinical and cost effectiveness of the Dutch versus the German rehabilitation approach following p-THA. It is a mutual project of the orthopedic departments of University Medical Center Groningen (UMCG) in the Netherlands and University Hospital for Orthopedics and Trauma Surgery Pius-Hospital, Medical Campus University of Oldenburg in Germany. Participating hospitals in the Netherlands were Ommelander Ziekenhuis Groningen (OZG) and Medical Center Leeuwarden (MCL), a large teaching hospital and a general hospital, respectively. German patients had surgery at Pius Hospital in Oldenburg and were inpatients at the rehabilitation center Reha-Zentrum am Meer in Bad Zwischenahn. All study-related measures were performed in accordance with the ethical principles formulated by the Declaration of Helsinki in its current version. In conformity, the study protocol was reviewed and ethical approval was provided by both the institutional review boards of both University Medical Center Groningen (METc2015/483) and Hannover Medical School (no. 2874–2015). Details of the study design are provided elsewhere [15].

### Study population

The following criteria were set up to determine patient eligibility. Inclusion criteria were employable patients aged 18–65, clinical evidence of hip OA according to Altman et al. [16], unilateral primary arthroplasty, and German patients’ agreement to do inpatient rehabilitation at the collaborative rehabilitation center. Exclusion criteria were medical conditions that disallow safe participation in a rehabilitation program, cognitive impairment, and inability to sufficiently read and understand German or Dutch (as applicable).

### Outcome measures

Preoperative demographic data, preoperative diagnosis, height, weight, body mass index (BMI), American Society of Anesthesiologists (ASA) classification, and perioperative/postoperative complications were recorded from electronic patient files. Measurements were taken preoperatively (T_0_) and at 4 weeks (T_1_), 12 weeks (T_2_), and 6 months (T_3_) postoperatively. To assess clinical effectiveness, satisfaction, functional status, and quality of life of p-THA patients were measured using patient self-reported questionnaires and objective functional measurements. To assess cost effectiveness a questionnaire was used at T_0_ and T_3_.

#### Primary outcome measure

Primary outcome was the patient acceptable symptom state (PASS), an instrument to measure a patient’s response to a treatment or intervention [17]. PASS is based on well-being or satisfaction with actual symptoms, expressed as the score on a patient-reported outcome measure beyond which patients consider themselves to be well [18, 19]. PASS scores were calculated from results on the subscale *function in activities of daily living (ADL)* of the Hip Disability and Osteoarthritis Outcome Score (HOOS). The HOOS is a self-reported disease-specific outcome measure consisting of five subscales: *pain* (10 items), *other symptoms* (5 items), *function in ADL* (17 items), *function in sports and recreational activities* (4 items), and *hip-related quality of life*(4 items). Standardized response options were given and each question was scored from 0 to 4 on a 5-point Likert Scale. A normalized score ranging from 0 to 100 was calculated for each subscale, with 0 indicating extreme symptoms and 100 indicating no symptoms. The German and Dutch versions are proven to be valid and reliable [20, 21]. To calculate the PASS scores of the HOOS an additional question was used asking patients about their actual satisfaction with their hip symptoms: *“If you were to spend the rest of your life with the hip symptoms you have now, how would you feel?”*This question was asked using a Likert Scale with four response options: very satisfied, somewhat satisfied, somewhat dissatisfied, very dissatisfied [18, 22]. The scores on this question were used to calculate the PASS, to distinguish between responders and non-responders.

#### Secondary outcome measures

To measure health-related quality of life, the Short Form 36 (SF-36) and the EuroQol 5 Dimensions 3 Level Questionnaire (EQ-5D-3L) were used. SF-36 is a widely used generic health status questionnaire, consisting of 36 questions organized into eight multi-item scales: physical functioning, role limitations due to physical problems, bodily pain, general mental health, social functioning, role limitations due to emotional problems, vitality, and general health perceptions. Each raw scale score is transformed into a linear 0–100 scale. A higher score represents less disability. The German- and Dutch-language versions are proven practical, reliable, and valid among general and chronically diseased populations [23, 24]. The EQ-5D-3L has five dimensions: mobility, self-care, usual activities, pain/discomfort, anxiety/depression. Each dimension is divided into three degrees of severity: no problems, some problems, and major problems. To calculate overall health status, responses were converted into an index following standardized specifications based on preference values drawn from respective country-specific general population samples [25]. For index calculation in the current study, the value set for the Netherlands was used for the Dutch patients and the value set for Germany was used for the German patients [25]. Actual quality of life was also identified on the EQ-5D-3L Visual Analog Scale (VAS) [26].

To assess functional status objectively, the Timed Up & Go Test (TUG) and the Five Times Sit-to-Stand Test (FTSST) were conducted. The TUG is considered a practical and reliable test to assess physical mobility [27]. The FTSST is a clinical test for assessing lower-extremity strength and balance, and is reliable and valid [28, 29]. To minimize learning effects as potential bias, the functional assessments were conducted three times as per instructions [30]. In-between trials, patients rested in a sitting position for 60 s. The mean for each test was calculated. The examiner was blinded to the results of previous measurement timepoints by using a blank report each time. To prevent bias, participants were not informed about their previous results.

#### Cost effectiveness

The economic evaluation was conducted in collaboration with the Patient Centered Health Technology Assessment Unit of UMCG. The aim was to obtain a first impression of the direct and indirect costs of postoperative care incurred in each of the two countries, since no pertinent data were previously available in the literature.

Direct costs post-treatment resulted from expenditures within the scope of care at the hospital, postoperative medical rehabilitation, other care providers (general practitioner, orthopedic technician, social worker), transportation, assistance at home, and other additional costs for the patients (over-the-counter medication, therapeutic services, remodeling measures). Indirect costs resulted from lost productivity due to incapacity for work.

Information on resource consumption was collected via a patient self-reported questionnaire, which is a generally accepted concept. Using this instrument, questions were asked about frequency and duration of medical visits and rehabilitation, use of therapeutic services, household chores, extra expenses incurred due to the hip complaints (e.g., for pain medication), time to return to work, extent of employment, need for professional retraining.

The monetary values on the Dutch side were obtained from the Dutch cost manual [31]. The monetary values for Germany were obtained as follows: Costs of inpatient medical rehabilitation were calculated using the daily rate of the rehabilitation facility multiplied by the patient's length of stay. Where exact monetary values could not be determined on the German side, data provided by the Organization for Economic Cooperation and Development (OECD) were used, always using the OECD healthcare expenditures of both countries as a share of the gross domestic product (GDP) to reflect the country-specific prices of healthcare facilities. German equivalents were calculated by multiplying Dutch costs by 1.06, as recommended by the OECD [32–34].

Productivity loss calculations followed the human capital approach, where missed working hours were multiplied by the respective hourly productivity costs over a time horizon of six months. Basis for the calculation of lost working time (in hours) was the number of working hours per week reported by each patient in the aforementioned questionnaire and the time of their return to work.

An overview of cost types, units and price per unit incurred in each country can be found in Additional file 1 and 2. All costs were calculated in euros in 2017 [35].

### Sample size

The sample size calculation was based on the PASS. The PASS is a novel approach to measure patients’ response to a treatment or intervention, and is an easy method to establish whether a patient has achieved therapeutic success [17]. According to Escobar et al., 70% of patients reach an acceptable-symptoms state at 12 weeks after p-THA. [17, 22] It was hypothesized that the German approach results in a larger proportion of patients with a positive PASS at 12 weeks following p-THA compared to the Dutch. It was stated that a difference of 20% between the German and Dutch samples in the proportion of patients with a positive PASS was considered clinically relevant [17, 36]. Hence based on the results of Escobar et al. a sample size of 60 patients in each subgroup (Germany/The Netherlands) was required to detect a 20% difference, with a power of 80% and a significance level of 0.05 [17, 22]. Considering a dropout rate of 20%, a final enrollment of 150 patients (75 Dutch and 75 German) was needed.

### Statistical analyses

Collected data were analyzed using SPSS software (V.23; IBM, Armonk, NY, USA). Main characteristics of both groups as well as intraoperative and postoperative complications were analyzed descriptively.

#### Primary Outcome Data Analysis

To distinguish patients who benefited from their p-THA and subsequent medical rehabilitation or follow-up (responders) from those who did not (non-responders), a receiver operating characteristic (ROC) curve was calculated as recommended in literature [17, 22]. The aim was to determine the optimal test cut-off value to distinguish between “responders” and “non-responders”. To this end, the PASS satisfaction question was first dichotomized. All patients who had ticked the PASS response option “very satisfied” or “somewhat satisfied” in the questionnaire were classified as “satisfied”; those who had indicated “somewhat dissatisfied” or “very dissatisfied” were classified as “dissatisfied”. This dichotomized variable was then used as anchor to calculate cut-off values on the subdomain function in ADL of the HOOS to determine the PASS score. A ROC analysis was conducted using the satisfied/unsatisfied variable as anchor. The optimal cut-off value was the one that maximized the sum of sensitivity and specificity. All patients whose HOOS subscale score was above the cut-off value calculated in this way were accordingly categorized as “responders” and all patients who were below were categorized as “non-responders”. Binary logistic regression (dependent variable: responder/non-responder; covariates: ASA score, educational level, sex, BMI), including calculation of odds ratios (OR) and corresponding 95% confidence interval (CI), was used to determine possible differences in the proportion of Dutch-to-German responders at T1, T2, and T3. The odds ratio represents the country-specific probability of being classified as a responder and is interpreted as statistically significant if the 95% CI does not include the value 1 [37].

#### Secondary Outcome Data analysis

Because data were collected at four timepoints, a multilevel analysis was performed to compare the secondary outcome measures. This made it possible to compare differences in both within-country and between-countries data at the different timepoints [37]. To increase estimated treatment effect’s precision, existing group differences in baseline values were adjusted. Means and 95% CI were then calculated. A p-value of.05 was considered statistically significant.

#### Cost Analysis

The respective mean values of all costs were calculated. Additionally, based on bootstrapping (5000 replications) the corresponding 95% CI were calculated. In a scenario analysis the correspondence between amount of work (in weekly hours) and productivity costs (per hour) in both countries was simulated in order to better compare the determined costs between the two countries. Information on resource use was collected via the aforementioned patient self-reported cost questionnaire.

## Results

### Participant characteristics

The study included 74 Dutch and 86 German patients. In each country 62 patients completed all the measurements. Reasons for dropout in the Netherlands were: unwillingness to continue participation (*n* = 3), additional surgery within six months (*n* = 2), unwillingness to perform functional tests (*n* = 2), death (*n* = 1), revision surgery of the same hip within six months (*n* = 1), cerebrovascular accident (*n* = 1), too much pain due to rheumatoid arthritis (*n* = 1), and too much pain in both hips (*n* = 1). Reasons for dropout in Germany were: unwillingness to continue participation (*n* = 7), revision surgery of the same hip within six months (*n* = 4), additional surgery within six months (*n* = 2), and cancelled surgery due to abnormal blood levels (*n* = 1). In addition, in Germany there were *n* = 10 screening failures (did not go to Bad Zwischenahn after inclusion).

Baseline characteristics of the patients are shown in Table 1. There were significant differences in educational level and ASA classification. A larger proportion of German patients (72.6%) had a lower educational level than Dutch patients (40.3%), and a larger proportion of Dutch patients (32.3%) had an ASA classification of III than German patients (11.3%).

**Table 1:** Baseline characteristics of the total study population and per country

	**Total (124)**	**Country**		***p-*** **value**
		**The Netherlands**	**Germany**	
		**(** ***n*** ** = 62)**	**(** ***n*** ** = 62)**	
Sex: female	65 (52.4)	36 (58.1)	29 (46.8)	.281^a^
Age (y; mean ± SD)	57.7 ± 6.0	58.8 ± 5.2	56.6 ± 6.6	.070^b^
BMI (kg/m^2^; mean ± SD)	29.5 ± 5.3	29.5 ± 5.3	29.5 ± 5.4	.816^b^
Educational level				**.002** ^a^
*Lower*	70 (56.5)	25 (40.3)	45 (72.6)	
*Secondary*	35 (28.2)	24 (38.7)	11 (17.7)	
*Higher*	19 (15.3)	13 (21.0)	6 (9.7)	
Living situation				1.000^a^
*Alone*	11 (8.9)	5 (8.1)	6 (9.7)	
*With partner and/or children*	113 (91.1)	57 (91.9)	56 (90.3)	
ASA classification				**.008**^**a**^
*I or II*	97 (78.2)	42 (67.7)	55 (88.7)	
*III*	27 (21.8)	20 (32.3)	7 (11.3)	
Comorbidities				.799^a^
*None*	18 (14.5)	8 (12.9)	10 (16.1)	
*One or more*	106 (85.5)	54 (87.1)	52 (83.9)	
Days in hospital (median (min–max))	9 (1—13)	2 (1—5)	11 (8—13)	**< .001**^**b**^

All data are presented as n (%) unless stated otherwise

Significant at 0.05 bolded

*Abbreviations*: *ASA* American Society of Anesthesiologists

^a^Fisher’s Exact Test

^b^Mann-Whitney U-test

Baseline scores on all outcomes are presented in Table 2. In the functional tests there were no significant differences at baseline between the two countries. For two of the five HOOS subscales Dutch patients scored significantly better at baseline than German patients. The same applies to five subscales of the SF-36 and the VAS score of the EQ-5D-3L.

**Table 2:** Outcome measures at baseline per country

	**The Netherlands**	**Germany**	***p*-value **^*e*^
	**(** ***n*** ** = 62)**	**(** ***n*** ** = 62)**	
***Functional measurements***			
TUG (sec)	11.3 (4.3)	12.1 (4.5)	.357
FTSST (sec)	19.7 (8.2)	18.8 (5.9)	.494
***HOOS***^*a*^			
Pain	41.1 (16.7)	35.0 (13.1)	**.028**
Other symptoms	39.4 (18.2)	36.4 (15.0)	.311
Function in ADL	40.2 (17.2)	35.2 (12.9)	.069
Function in sport & recreational activities	19.6 (18.0)	15.3 (13.0)	.130
Hip-related QoL	24.9 (15.5)	18.6 (11.7)	**.012**
***SF-36***^*b*^			
Physical functioning	32.7 (16.4)	21.7 (14.9)	**< .001**
Role limitations: physical	19.4 (33.7)	14.5 (25.0)	.366
Bodily pain	36.1 (14.4)	21.3 (13.6)	**< .001**
General health perception	64.2 (18.5)	55.8 (19.2)	**.014**
Vitality	52.3 (17.2)	42.6 (17.0)	**.002**
Social functioning	69.2 (27.4)	63.7 (27.6)	.273
Role limitations: emotional	73.1 (41.7)	70.4 (44.0)	.728
General mental health	76.5 (15.8)	64.9 (19.9)	**< .001**
***EQ-5D-3L***			
Index score ^*c*^	0.6 (0.3)	0.6 (0.3)	.769
VAS score ^*d*^	64.9 (15.5)	51.3 (21.3)	**< .001**

All data are presented as mean (SD)

Significant at 0.05 bolded

*Abbreviations*: *ADL* activities of daily living, *EQ-5D-3L* EuroQol 5 Dimensions 3 Level Questionnaire, *FTSST* Five Times Sit-to Stand Test, *HOOS* Hip disability and Osteoarthritis Outcome Score, *QoL* quality of life, *SF-36* 36-item Short Form Health Survey, *TUG* Timed Up & Go test, *VAS* visual analog scale

^a^Scale scores range 0–100 (0 = extreme symptoms, 100 = no symptoms)

^b^Scale scores range 0–100 (higher score = better perceived health or functioning)

^c^Scale scores range 0–1 (higher score = better health-related quality of life)

^d^Scale scores range 0–100 (higher score = better health-related quality of life)

^e^Results of Independent t-test

### Satisfaction based on the PASS

The percentages of Dutch and German patients who scored “satisfied” on the PASS are shown in Table 3. The cut-off values on the HOOS subscale *function in ADL* were calculated with the PASS; 12 weeks postoperatively this cut-off value was 81.6, with 65.6% of German patients and 47.5% of Dutch patients scoring above the cut-off value.

**Table 3:** Percentages of satisfied patients

	**4 weeks**	**12 weeks**	**6 months**
**Function in ADL**			
*Cut-off value, ROC curve*	68.4	81.6	86.0
*AUC (95% CI)*	0.74 (0.65–0.83)	0.85 (0.75–0.95)	0.77 (0.63–0.91)
**Responder (%, Netherlands/Germany)**	40.3%/50.0%	47.5%/65.6%	58.1%/69.4%
**Odds ratio (95% CI)**	1.53 (0.75–3.12)	**2.10 (1.01–4.36)**	1.64 (0.78–3.42)
**Odds ratio adjusted (95% CI)**^**a**^	1.16 (0.52–2.57)	2.15 (0.94–4.91)	**2.44 (1.01–5.89)**

Percentages of satisfied patients (those patients who were “very satisfied” or “somewhat satisfied”), cut-off values of subdomain function in ADL of the HOOS by ROC curve, and percentages of patients who were “responders” (those patients scoring above the cut-off value), at 4 weeks (T), 12 weeks (T2), and 6 months (T3). The odds of being a responder were calculated with a logistic regression

Significant at 0.05 bolded

*Abbreviations*: *ADL *activities of daily living, *AUC* area under the curve, *CI* confidence interval, *PASS* patient acceptable symptom state, *ROC*: receiving operating characteristics

^a^Adjusted for gender, BMI, educational level, and ASA classification

The results of the binary logistic regressions, also displayed in Table 3, show that German patients have 2.10 higher chances of becoming responders to rehabilitation 12 weeks postoperatively than Dutch patients. When taking age, BMI, educational level, and ASA classification into consideration, German patients still appear to have higher chances, albeit no longer significant, of becoming responders to rehabilitation than Dutch patients (OR = 2.15). And yet six months postoperatively German patients appeared to have significantly higher chances of becoming responders to rehabilitation, after adjusting for age, BMI, educational level, and ASA classification (OR = 2.44).

### Functional and self-reported outcomes

Table 4 presents the outcomes of the functional tests and self-reported questionnaires, adjusted for educational level, ASA classification, and as needed for baseline. Significant differences were found on the FTSST at 4 weeks and 12 weeks postoperatively between the two countries, favoring Germany. No significant differences were found for the TUG.

**Table 4:** Within-measurements comparison between the countries adjusted for baseline differences

	*Netherlands*	*Germany*	*95% CI*^*e*^	
	*Mean (95% CI)*	*Mean (95% CI)*	*Lower*	*Upper*
**Timed Up & Go test**				
***T0***	11.5 (10.6–12.3)	12.1 (11.1–13.1)	-1.9	0.6
***T1***	13.3 (12.5–14.2)	13.0 (12.0–14.0)	-0.9	1.6
***T2***	9.5 (8.6–10.3)	9.0 (8.0–10.0)	-0.8	1.7
***T3***	8.5 (7.7–9.4)	8.2 (7.2–9.2)	-0.9	1.5
**Five Times Sit-to Stand Test**				
***T0***	19.6 (18.4–20.8)	18.1 (16.7–19.6)	-0.3	3.2
***T1***	17.8 (16.6–19.0)	16.0 (14.6–17.4)	**0.1**	**3.5**
***T2***	14.8 (13.6–16.0)	12.8 (11.4–14.2)	**0.2**	**3.7**
***T3***	13.5 (12.3–14.7)	12.3 (10.9–13.7)	-0.5	2.9
**HOOS**^**a**^				
*Pain*				
***T0***	41.5 (37.7–45.3)	36.7 (32.2–41.1)	-0.6	10.3
***T1***	74.5 (70.7–78.3)	78.1 (73.7–82.5)	-9.0	1.9
***T2***	84.5 (80.6–88.3)	89.6 (85.2–94.0)	-10.6	0.3
***T3***	87.9 (84.1–91.7)	93.1 (88.7–97.5)	-10.6	0.3
*Other symptoms*				
***T0***	39.8 (35.9–43.6)	37.6 (33.2–42.0)	-3.3	7.6
***T1***	67.2 (63.4–71.1)	75.1 (70.7–79.5)	**-13.4**	**-2.4**
***T2***	77.4 (73.5–81.3)	83.0 (78.6–87.4)	**-11.1**	**-0.1**
***T3***	80.6 (76.7–84.4)	87.2 (82.8–91.6)	**-12.1**	**-1.1**
*Function in ADL*				
***T0***	40.5 (36.6–44.4)	36.2 (31.6–40.7)	-1.3	9.9
***T1***	63.5 (59.6–67.5)	69.1 (64.6–73.7)	**-11.2**	**0.0**
***T2***	77.9 (73.9–81.8)	85.0 (80.5–89.6)	**-12.8**	**-1.6**
***T3***	84.1 (80.2–88.0)	89.9 (85.4–94.5)	**-11.4**	**-0.3**
*Function in sports and recreational activities*				
***T0***	19.3 (13.8–24.8)	14.8 (8.4–21.2)	-3.3	12.3
***T1***	37.3 (31.7–42.9)	40.6 (34.2–47.0)	-11.2	4.7
***T2***	59.7 (54.1–65.2)	64.1 (57.7–70.5)	-12.3	3.4
***T3***	67.9 (62.4–73.4)	75.1 (68.7–81.5)	-15.0	0.6
*Hip-related QoL*				
***T0***	25.0 (20.4–29.6)	18.6 (13.3–23.8)	-0.1	12.9
***T1***	48.8 (44.2–53.4)	50.8 (45.5–56.1)	-8.6	4.5
***T2***	67.0 (62.4–71.6)	67.8 (62.5–73.0)	-7.4	5.7
***T3***	72.3 (67.7–76.8)	77.0 (71.7–82.2)	-11.2	1.8
**SF-36**^**b**^				
*Physical functioning*				
***T0***	30.0 (25.4–34.5)	24.0 (18.7–29.2)	-0.6	12.6
***T1***	39.0 (34.5–43.6)	47.3 (42.0–52.6)	**-14.9**	**-1.7**
***T2***	62.3 (57.7–66.9)	73.2 (67.9–78.4)	**-17.5**	**-4.3**
***T3***	69.9 (65.3–74.5)	81.1 (75.8–86.3)	**-17.7**	**-4.7**
*Role limitations: physical*				
***T0***	21.0 (12.0–29.9)	16.4 (6.3–26.6)	-8.2	17.2
***T1***	12.9 (4.0–21.9)	21.8 (11.7–32.0)	-21.6	3.9
***T2***	49.2 (40.2–58.2)	55.7 (45.5–65.9)	-19.4	6.3
***T3***	68.1 (59.2–77.0)	81.4 (71.2–91.5)	-25.9	0.6*
*Bodily pain*				
***T0***	34.0 (29.0–39.0)	25.3 (19.8–30.9)	**1.6**	**15.8**
***T1***	43.4 (38.4–48.4)	52.4 (46.8–58.0)	**-16.2**	**-1.9**
***T2***	61.9 (56.9–67.0)	77.8 (72.2–83.4)	**-23.0**	-8.7*
***T3***	71.6 (66.6–76.6)	86.6 (81.0–92.1)	**-22.1**	-7.9*
*General health perception*				
***T0***	60.5 (56.7–64.3)	57.3 (52.9–61.7)	-2.2	8.7
***T1***	68.4 (64.6–72.1)	68.9 (64.4–73.3)	-5.9	5.0*
***T2***	67.5 (63.7–71.3)	73.0 (68.6–77.4)	**-10.9**	0.0*
***T3***	67.6 (63.9–71.4)	72.3 (68.0–76.7)	-10.1	0.7*
*Vitality*				
***T0***	49.7 (45.9–53.6)	45.6 (41.2–50.1)	-1.5	9.6
***T1***	54.2 (50.4–58.1)	57.7 (53.2–62.1)	-9.0	2.1
***T2***	63.7 (59.8–67.6)	69.0 (64.6–73.5)	-10.9	0.2
***T3***	63.5 (59.6–67.3)	74.6 (70.2–79.0)	**-16.7**	-5.6*
*Social functioning*				
***T0***	69.4 (63.5–75.3)	64.5 (57.6–71.4)	-3.5	13.3
***T1***	57.9 (52.0–63.8)	72.3 (65.4–79.2)	**-22.8**	-5.9*
***T2***	83.6 (77.7–89.5)	90.1 (83.4–97.0)	-15.0	1.9
***T3***	87.7 (81.9–93.6)	92.3 (85.5–99.2)	-13.0	3.8
*Role limitations: emotional*				
***T0***	75.3 (65.2–85.4)	75.9 (64.2–87.6)	-15.0	13.8
***T1***	58.1 (48.0–68.2)	72.8 (61.0–84.6)	**-29.2**	-0.3*
***T2***	80.1 (70.1–90.3)	92.0 (80.3–103.7)	-26.3	2.6*
***T3***	84.4 (74.3–94.5)	96.3 (84.6–108.0)	-26.3	2.5*
*General mental health*				
***T0***	72.9 (69.6–76.2)	68.4 (64.7–72.0)	-0.1	9.2
***T1***	74.8 (71.5–78.1)	76.6 (72.9–80.3)	-6.5	2.8
***T2***	81.8 (78.5–85.1)	82.6 (79.0–86.3)	-5.5	3.8
***T3***	78.6 (75.4–81.9)	85.2 (81.6–88.9)	**-11.2**	**-1.9**
**EQ-5D-3L**				
*Index score*^*c*^				
***T0***	0.6 (0.5–0.6)	0.6 (0.6–0.7)	-0.1	0.0
***T1***	0.7 (0.7–0.8)	0.9 (0.8–0.9)	**-0.2**	**-0.1**
***T2***	0.8 (0.8–0.9)	0.9 (0.9–1.0)	**-0.2**	**-0.1**
***T3***	0.8 (0.8–0.9)	1.0 (0.9–1.0)	**-0.2**	**-0.1**
*VAS score*^*d*^				
***T0***	61.7 (58.4–65.0)	53.2 (49.5–57.0)	**3.7**	**13.2**
***T1***	68.1 (64.8–71.4)	73.0 (69.3–76.8)	**-9.7**	**-0.2**
***T2***	73.9 (70.6–77.3)	82.7 (79.0–86.4)	**-13.5**	**-4.0**
***T3***	74.6 (71.3–77.9)	84.8 (81.0–88.5)	**-14.9**	**-5.4**

All data are presented as mean (95% CI)

Significant at 0.05 bolded

*Abbreviations*: *ADL* activities of daily living, *EQ-5D-3L* EuroQol 5 Dimensions 3 Level Questionnaire, *FTSST* Five Times Sit-to Stand Test, *HOOS* Hip disability and Osteoarthritis Outcome Score, *QoL* quality of life, *SF-36* 36-item Short Form Health Survey, *TUG* Timed Up & Go test, *VAS* visual analog scale

^a^Scale scores range 0–100 (0 = extreme symptoms, 100 = no symptoms)

^b^Scale scores range 0–100 (higher score = better perceived health or functioning)

^c^Scale scores range 0–1 (higher score = better health-related quality of life)

^d^Scale scores range 0–100 (higher score = better health-related quality of life)

^e^Multilevel analysis

^*^Result is above the value of the minimum clinically relevant difference (MCID). MCIDs of each HOOS subscales are 9 [38]. MCIDs of the SF36 subscales are: 20.40 (physical functioning), 10.78 (role limitations: physical), *14.67* (bodily pain), 0.40 (general health perception), 10.14 (vitality), 8.63 (social functioning), 6.45 (role limitations: emotional), 8.99 (general mental health) [39]. MCIDs of the EQ-5D-3L are: 0.31 (index value) and 23 (VAS value) [40]

Significant differences were found in the self-reported questionnaires favoring German patients on the subscales *function in ADL* and *other symptoms* of the HOOS. Significant postoperative differences appeared at 4 weeks and were still present at 6 months. On the other subscales of the HOOS differences were likewise found favoring German patients, although Dutch patients fared better at baseline.

On the health-related QoL questionnaires German patients scored significantly better on most subscales of the SF-36, especially on physical functioning and bodily pain, resembling the results on the HOOS. On both subscales of the EQ-5D-3L significant postoperative differences appeared at 4 weeks and were still present at 6 months, favoring German patients.

### Cost effectiveness – a first impression

The average total costs incurred per country are shown in Table 5. The German medical rehabilitation approach caused additional costs of €7032 (+ 45%) compared to the Dutch approach. Looking only at the costs in the subgroup of employed patients (Table 6), the German approach, at €4243, is still 20% more expensive than the Dutch approach. This can be explained by the larger number of therapeutic interventions following p-THA on the German side, and by the fact that loss of productivity resulting from inability to work is significantly higher in Germany than in the Netherlands.

**Table 5:** Cost effectiveness of all patients (postoperatively), mean (95% CI)

	**Netherlands (*n* = 61**^**a**^**)**	**Germany (** ***n*** ** = 62)**	**Difference**
**Direct costs**			
**Medical costs**			
***Outpatient care***			
Orthopedic surgeon	€ 83	€ 48	€ -35
Rehabilitation department	€ 4	€ 7	€ 3
Psychiatrist	€ 0	€ 2	€ 2
Other	€ 13	€ 0	€ -13
***Total***	**€ 100 (82–142)**	**€ 57 (24–58)**	**€ -43 (-107– -37)**
**Postoperative rehabilitation**			
Inpatient rehabilitation	_-_	€ 3054	€ 3054
Medical training therapy	_-_	€ 158	€ 158
Sports rehabilitation	_-_	€ 26	€ 26
Physiotherapy	€ 552	€ 646	€ 94
***Total***	**€ 552 (330–594)**	**€ 3898 (3522–3999)**	**€ 3346 (3028–3574)**
**Other care providers**			
General practitioner	€ 3	€ 40	€ 37
Orthopedic technician	€ 4	€ 2	€ -2
Social worker	€ 0	€ 4	€ 4
***Total***	**€ 5 (1–10)**	**€ 47 (22–68)**	**€ 42 (17–63)**
***Extra Expenses***^***b***^			
Medication/Therapy	€ 66	€ 32	€ -34
Other	€ 52	€ 246	€ 194
***Total***	**€ 120 (23–245)**	**€ 286 (112–470)**	**€ 166 (-57– + 375)**
**Non-medical costs**			
**Travel expenses**			
***Total***	**€ 18 (11–21)**	**€ 63 (55–75)**	**€ 46 (38–60)**
**Household help**			
Alpha help	€ 21	€ 26	€ 5
Paid by other sources	€ 40	€ 44	€ 4
***Total***	**€ 61 (4–144)**	**€ 72 (0–156)**	**€ 11 (-104– + 114)**
**Indirect costs**			
**Paid work**			
Productivity loss	€ 7658 (4828–10194)	€ 11122 (9254–15541)	€ 3464 (731–9012)
**Total**	**€ 8514**	**€ 15545**	**€ 7031 (45%)**

^a^One Dutch patient did not want to complete the cost questionnaire, hence the Dutch sample here is *n* = 61 instead of *n* = 62

^b^“Extra expenses” was an open-ended question in the questionnaire that asked participants about any additional expenses they had incurred in connection with the hip problem, like medications or therapeutic lines, unexpected large expenses, cancellation of a vacation due to surgery date, necessary home remodeling

**Table 6:** Cost effectiveness of working patients (postoperatively), mean (95% CI)

	**Netherlands (** ***n*** ** = 35)**	**Germany (** ***n*** ** = 42)**	**Difference**
**Direct costs**			
**Medical costs**			
***Outpatient care***			
Orthopedic surgeon	€90	€53	€-37
Rehabilitation department	€3	€9	€6
Psychiatrist	€0	€0	€0
Other	€3	€0	€-3
***Total***	**€95 (54–145)**	**€62 (36–92)**	**€-33 (-90– + 20)**
**Postoperative rehabilitation**			
Inpatient rehabilitation	_-_	€3069	€3069
Medical training therapy	_-_	€159	€159
Sports rehabilitation	_-_	€18	€18
Physiotherapy	€593	€592	€-1
***Total***	**€593 (421–777)**	**€3843 (3652–4054)**	**€3250 (2997–3521)**
**Other care providers**			
General practitioner	€4	€54	€50
Orthopedic technician	€1	€2	€1
Social worker	€0	€5	€5
***Total***	**€5 (0–12)**	**€61 (32–95)**	**€56 (26–91)**
***Extra Expenses***^***a***^			
Medication/Therapy	€44	€38	€-6
Other	€43	€243	€200
***Total***	**€87 (9–192)**	**€280 (87–572)**	**€193 (-36– + 503)**
**Non-medical costs**			
**Travel expenses**			
***Total***	**€15 (11–19)**	**€62 (50–74)**	**€47 (35–60)**
**Household help**			
Alpha help	€7	€38	€31
Paid by other sources	€70	€65	€-5
***Total***	**€78 (0–203)**	**€104 (7–236)**	**€27 (-134– + 182)**
**Indirect costs**			
**Paid work**			
Productivity loss	€ 15622 (12183–19026)	€ 16326 (12682–20242)	€ 703 (-4313– + 5986)
**Total**	**€ 16495**	**€ 20738**	**€ 4243 (20%)**

^a^“Extra expenses” was an open-ended question in the questionnaire that asked participants about any additional expenses they had incurred in connection with the hip problem, like medications or therapeutic lines, unexpected large expenses, cancellation of a vacation due to surgery date, necessary home remodeling

## Discussion

Primary aim of this study was to compare clinical effectiveness between the common medical rehabilitation approach after p-THA in Germany and the common approach in the Netherlands for working-age patients against the background of the prognosis that the majority of p-THA patients will be from the working-age population [41]. Secondary objective was to gain a first impression of the costs incurred during treatment. For country-specific p-THA post-treatment differences, it was hypothesized that the much more comprehensive medical rehabilitation as practiced in Germany would lead to higher patient satisfaction and better functional outcomes than the leaner Dutch approach without medical rehabilitation. In addition, the German procedure was assumed to be more cost-effective in the medium term than the Dutch procedure, even though it appears much more expensive at first glance.

Looking at clinical effectiveness, data collected in the present study confirm the hypothesis that the German procedure results in a significantly higher proportion of patients (65.6% of German vs. 47.5% of Dutch participants) who are satisfied 12 weeks after p-THA and who have benefited from surgery and subsequent follow-up treatment. At 6 months postoperatively, German patients were still more satisfied than Dutch patients (69.4% vs. 58.1%). Overall, German patients are twice as likely to benefit from surgery and related medical rehabilitation measures and thus achieve greater satisfaction.

The German cohort scored significantly better on self-reported function. Some of these differences were clinically relevant, especially on the subdomains of the SF-36, namely *role limitations due to physical problems, bodily pain, general health perception, social functioning, role limitations due to emotional problems*, and *vitality*. It also seems noteworthy that German patients scored significantly better on pain relief following p-THA surgery and an intense medical rehabilitation. Hence one may speculate that the German approach may lead to psychological benefits that in turn could impact patients’ long-term recovery, well-being, and thus quality of life. On the functional tests a significant difference was found on the FTSST at 4 and 12 weeks postoperatively. For the remaining timepoints and TUG German patients tended to score better.

Füssenich et al. also compared the usual p-THA treatment in the Netherlands to the German approach [42]. In contrast to the current study, they found no significant between-countries differences in terms of patient satisfaction and subjectively perceived hip function. This may be due to the different design of the two studies: Füssenich et al. included p-THA patients of all age groups. In addition, postoperative measurements started only after 6 months, followed by a follow-up after 12 months. The development of the rehabilitation course in the first postoperative months was not explicitly investigated. However, especially for working-age patients the early rehabilitation phase is imperative for their return to working life. We showed that German patients return 1.9 weeks earlier to work than Dutch patients. Füssenich et al. only investigated direct medical costs and not indirect costs, which are particularly relevant for the working patient population. Also, in contrast to the present study, they did not include objective functional measurements of the hip.

In terms of cost analyses, the present study should and can only provide a first impression. Cost comparisons are challenging because of the substantial between-country differences in socioeconomic context and essential structures of the healthcare system. On one side of the border, for example, there are certain specialists, medical professionals and/or therapeutic approaches that do not exist in the other country (e.g., outpatient specialists, land-based and/or water-based medical rehabilitation). For this reason, all information on resource use was collected via a patient self-reported questionnaire, which was partly adapted to the respective system. Where precise monetary evaluation rates could not be determined on the German side, data from the OECD were tapped, with the OECD health expenditure of both countries used as a share of GDP in each case to reflect country-specific prices of healthcare facilities. A more accurate impression could only be obtained if all costs were available without restriction in both countries.

The German approach appears considerably more expensive than the Dutch, given the much more intensive multimodal and interdisciplinary medical p-THA rehabilitation in Germany. The German approach is also more expensive given the higher productivity losses due to work absenteeism. This can be explained by a higher amount of regular weekly working hours and higher wage costs in Germany. Overall, considering this study’s total population, medical rehabilitation costs in connection with p-THA are almost twice as high in Germany (45%) as in the Netherlands. However, when comparing only patients who are actively employed a more balanced picture emerges where the costs in Germany are only about 20% higher than in the Netherlands.

As mentioned, the average weekly working hours of German patients are higher than those of Dutch patients, as is the hourly wage in Germany (Additional file 3). As a result, a German employee’s incapacity to work costs more than that of a Dutch employee for an equivalent period and the productivity loss of German patients is higher. However, when the missed work hours are converted into missed weeks, German patients return to work two weeks earlier than Dutch patients after p-THA, with a productivity loss in Germany of €2471. This amount largely compensates for the costs incurred by the 3-week inpatient stay for medical rehabilitation at a dedicated facility (average costs Reha-Zentrum am Meer: €3069). For an even better comparison of the costs incurred, an additional scenario analysis was conducted (Additional file 4) simulating a match of all variables between the groups to be compared. In this case it was assumed that German patients worked the same number of weekly hours as Dutch patients and that the productivity costs per hour were similar in both countries. The costs for postoperative medical rehabilitation in Germany are still 8% higher (€1355) even under such postulated identical conditions, but the cost picture is much more differentiated than before.

For clinical practice, the present study’s results suggest that it must be questioned whether it wouldn’t be useful for the Netherlands to adopt the German approach to medical rehabilitation after p-THA, or at least aspects of it. In the 1970s, when the first p-THAs were implanted, most patients were already of advanced retirement age and had a lower activity level [43]. Due to higher life expectancy, later retirement, more frequent p-THA implantation also in employable younger patients due to early osteoarthritis (e.g., caused by obesity), and technical and surgical progress [44, 45], the patient group is more heterogeneous nowadays. In this context it can be argued that a one-size-fits-all approach to rehabilitation is no longer appropriate. The current p-THA post-treatment approach may be sufficient for generally inactive Dutch patients (e.g., in advanced retirement), yet may not hold for the growing group of younger patients who need to return to the labor market with their artificial hip joint. This group of patients would probably benefit from more intensive medical rehabilitation according to the German model.

The present study must be evaluated, given its limitations. Among these is that the concept of common p-THA follow-up in the Netherlands is difficult to define, as implementation of physiotherapeutic measures can vary widely from patient to patient. Both the Dutch Orthopaedic Association and the Royal Dutch Physiotherapy Association recommend continuous physiotherapy follow-up after p-THA and hospital discharge to correct remaining dysfunctions; increase muscle strength, mobility, and stability; harmonize gait; and minimize limitations in activities of daily living [46, 47]. The choice of modalities in this process and their extent and frequency remain open. In addition, postoperative physiotherapeutic treatment is essentially not covered by Dutch basic health insurance [9]. It must be financed by supplementary insurance or privately, so the number of therapy sessions and their content vary considerably. Dutch patients who received more physiotherapy may be similar to German patients in terms of hip function and subjective feelings. To investigate this, we conducted a sensitivity analysis between the two groups, considering on the Dutch side only those patients (*n* = 28) who had used postoperative physiotherapy more frequently than average (≥ 15 treatments). However, even in such an adjusted analysis the same picture emerged: the difference between German and Dutch patients with positive PASS remained constant (± 20%), favoring German patients.

Another limitation could be that on the German side only those patients were included who agreed at study enrollment to have their rehabilitation treatment in the cooperating rehabilitation center, which represents a possible selection bias. However, it should be noted that the evidence-based rehabilitation standards defined for p-THA by the DRV apply equally to all rehabilitation facilities and implementation is standardized. In this respect, it can be assumed that the effects would not have been substantial even if such a bias had existed.

Another limitation is that no comparison between German and Dutch patients was possible regarding payment of wage replacement benefits, since only a minority of German patients could provide information on receipt of such benefits and Dutch patients were not asked about their income during their absence from work. The cost comparison is limited by the fact that early rehabilitation services at the hospital were not specifically considered in the present cost analysis. German patients stay longer at the hospital postoperatively and thus also receive physiotherapeutic services longer in the sense of early rehabilitation compared to the Dutch.

Future studies should follow p-THA patients from both countries and systems over a longer period to evaluate the long-term effects of the respective follow-up treatments on functional capacity, activities, and participation. This should include an examination of the influence of cultural differences on rehabilitation outcomes. Since the economic comparison of the medical rehabilitation approaches in the current study can only give a first impression, it would also be important to conduct studies that shed further light on the economic aspect. Such studies should include larger cohorts and follow them over a longer period.

## Conclusions

This study identified that a more intense aftercare following p-THA as performed in Germany is clinically advantageous. From a cost-effectiveness perspective comparisons are less straightforward, as the socioeconomic context differs between the two countries. The results nonetheless give food for thought as to whether the German approach, or at least aspects of it, could be beneficial for the expanding group of employable patients in the Netherlands.

## Supplementary Information

**Additional file 1.** Types of costs, unit, and unit prices per country.

**Additional file 2.** Average distance from household to care venue.

**Additional file 3.** Additional insight into the working situation of the working patient group, mean (95% CI).

**Additional file 4.** Scenario analysis of working patients.

## Data Availability

Available upon request from the corresponding author (Martin Stevens).

## Abbreviations

ADL: Activities of Daily Living
ASA: American Society of Anesthesiologists
BMI: Body Mass Index
CI: Confidence Interval
DRV: German Pension Insurance
EQ-5D-3L: EuroQol 5 Dimensions 3 Level Questionnaire
FTSST: Five Times Sit-to-Stand Test
GDP: Gross Domestic Product
HOOS: Hip Disability and Osteoarthritis Outcome Score
MCL: Medical Center Leeuwarden
OA: Osteoarthritis
OECD: Organization for Economic Cooperation and Development
OR: Odds Ratios
OZG: Ommelander Ziekenhuis Groningen
PASS: Patient Acceptable Symptom State
p-THA: Primary Total Hip Arthroplasty
QoL: Quality of Life
ROC curve: Receiver Operating Characteristic curve
SF-36: Short Form 36
THA: Total Hip Arthroplasty
TUG: Timed Up & Go Test
UMCG: University Medical Center Groningen
VAS: Visual Analog Scale
