# Supplementary material for: Effectiveness of rehabilitation for working-age patients after a total hip arthroplasty: a comparison of usual care between the Netherlands and Germany
Source: BMC Musculoskelet Disord. 2023 Jun 27;24:525. doi: 10.1186/s12891-023-06654-w (PMC10294515; doi:10.1186/s12891-023-06654-w)
Supplement: Supplementary file 1 — Additional file 1. Types of costs, unit, and unit prices per country. [file 12891_2023_6654_MOESM1_ESM.docx]

Additional file 1. Types of costs, unit, and unit prices per country.

|  | |  | **Unit** | **Unit price (NL)^a^** | **Unit price (DE)^b^** |
| --- | --- | --- | --- | --- | --- |
| **Direct costs** | | |  |  |  |
| **Medical costs** | | |  |  |  |
|  | ***Outpatient care*** | |  |  |  |
|  | Outpatient clinic visit, general hospital | | visit | € 83.95 | - |
|  | Outpatient clinic visit, academic hospital | | visit | € 171.05 | - |
|  | Orthopedic specialist | | visit | - | € 27.50 ^d^ |
|  | Rehabilitation physician | | visit | - | € 27.50 ^d^ |
|  | Consult with psychiatrist | | visit | € 98.64 | € 104.91 |
|  | ***Postoperative rehabilitation*** | |  |  |  |
|  | Inpatient rehabilitation | | session | - | € 143.55 ^c^ |
|  | Medical training therapy | | session | - | € 255 ^c^ |
|  | *Rehasport* | | session | - | € 5.25 ^c^ |
|  | Aquatic training | | session | - | € 6.11 ^c^ |
|  | Physiotherapy | | session | € 34.63 | € 36.83 |
|  | ***Other care providers*** | |  |  |  |
|  | General practitioner, standard consultation | | visit | € 34.63 | € 36.83 |
|  | Orthopedic technician/podologist | | visit | € 34.63 | € 36.83 |
|  | Consult with social worker | | visit | € 68.21 | € 72.54 |
| ***Extra Expenses^e^*** | | |  |  |  |
| **Non-medical costs** | | |  |  |  |
|  | ***Travel expenses*** | |  |  |  |
|  | Car | | km | € 0.20 | € 0.21 |
|  | Parking | | visit | € 3.15 | € 3.35 |
|  | Public transport | | km | € 0.20 | € 0.21 |
|  | On foot or by bike | | km | - | - |
|  | ***Household help*** | |  |  |  |
|  | Domestic work at home (Alpha help) | | hour | € 20.99 | € 22.32 |
|  | Domestic support at home (Help paid otherwise) | | hour | € 24.14 | € 25.67 |
|  | | |  |  |  |
| **Indirect costs** | | |  |  |  |
|  | **Paid work** | |  |  |  |
|  | Productivity costs (women) | | hour | € 33.16 | € 35.27 |
|  | Productivity costs (men) | | hour | € 39.77 | € 42.30 |

Abbreviations: DE: Germany; NL: The Netherlands

^a^ Dutch cost manual (2014) [31] multiplied by 1.04 (inflation increase) to calculate costs in 2017

^b^ Some Dutch costs were multiplied by 1.06 (index score) to calculate German costs, reference: Organisation for Economic Co-operation and Development (OECD) [32]

^c^ The cooperating rehabilitation center´s controlling department^d^ Bock et al (2011) [47] multiplied by 1.08 (inflation increase) to calculate costs in 2017

^e^ “Extra expenses” was an open-ended question in the questionnaire that asked participants about any additional expenses they may have incurred related to the hip problem; e.g., additional expenses for medications or therapeutic lines, unexpected large expenses, cancellation of a vacation due to surgery date, necessary home remodeling.
