# Supplementary material for: Effectiveness of rehabilitation for working-age patients after a total hip arthroplasty: a comparison of usual care between the Netherlands and Germany
Source: BMC Musculoskelet Disord. 2023 Jun 27;24:525. doi: 10.1186/s12891-023-06654-w (PMC10294515; doi:10.1186/s12891-023-06654-w)
Supplement: Supplementary file 2 — Additional file 2. Average distance from household to care venue. [file 12891_2023_6654_MOESM2_ESM.docx]

Additional file 2. Average distance from household to care venue

|  | **The Netherlands^a^** | **Germany^b^** |
| --- | --- | --- |
| General practitioner | 1.1 km | 2.1 km |
| Orthopedic technician | 7.0 km | 13.4 km |
| Social worker | 2.2 km | 4.2 km |
| Physiotherapist | 2.2 km | 4.2 km |

^a^ Dutch cost manual;

^b^ The Dutch distance was multiplied by 1.92* to calculate the German distance.

*The questionnaire asked where the orthopedic/rehabilitation physician/outpatient. Since the patients' place of residence was known, the distances could be calculated accordingly. For the German patients, this was a longer distance than for the Dutch patients by a factor of 1.92. The distance to the physiotherapist/general practitioner/etc. was not asked in the questionnaire. In the Netherlands, however, the average distance from a patient's home to the physiotherapist/general practitioner/etc. is known (see Dutch cost manual). To calculate the German distance to these facilities, the Dutch one was multiplied by a factor of 1.92.
