# Supplementary material for: Effectiveness of rehabilitation for working-age patients after a total hip arthroplasty: a comparison of usual care between the Netherlands and Germany
Source: BMC Musculoskelet Disord. 2023 Jun 27;24:525. doi: 10.1186/s12891-023-06654-w (PMC10294515; doi:10.1186/s12891-023-06654-w)
Supplement: Supplementary file 3 — Additional file 3. Additional insight into the working situation of the working patient group, mean (95% CI). [file 12891_2023_6654_MOESM3_ESM.docx]

Additional file 3. Additional insight into the working situation of the working patient group, mean (95% CI).

|  | **The Netherlands (n=35)** | **Germany (n=42)** | **Difference** |
| --- | --- | --- | --- |
| Missed hours | 412.1 | 421.8 | 9.71 |
| Missed productivity costs | € 15622 (12183 - 19026) | € 16326 (12682 - 20242) | € 703 (-4313 - + 5986) |
| Working hours (per week) | 28.1 | 33.6 | 19.33 % (Index: 1.19) |
| Missed weeks | 14.7 | 12.6 | 1.9 |
| Productivity costs per week | €1065 | €1301 | €236 |
| Productivity loss in **2.3** weeks | €2023 | €2471 | €488 |
| Still not working after 6 months | 8 Patients | 6 Patients |  |
|  | (male: 5/female: 3) | (male: 3/female: 3) |  |
