# Supplementary material for: Effectiveness of rehabilitation for working-age patients after a total hip arthroplasty: a comparison of usual care between the Netherlands and Germany
Source: BMC Musculoskelet Disord. 2023 Jun 27;24:525. doi: 10.1186/s12891-023-06654-w (PMC10294515; doi:10.1186/s12891-023-06654-w)
Supplement: Supplementary file 4 — Additional file 4. Scenario analysis of working patients. [file 12891_2023_6654_MOESM4_ESM.docx]

Additional file 4. Scenario analysis of working patients.

|  | **The Netherlands (n=35)** | **Germany (n=42)** | **Difference** |
| --- | --- | --- | --- |
| **Sum of direct costs** | **€873** | **€4412** | **€3539** |
| Missed hours | 412.1 | 354.5 | -57.6 |
| Productivity loss | € 15622 | €13438 | €-2002 |
| **Total** | **€16495** | **€17850** | **€-1355 (8 %)** |
| Working hours (per week) | 28.1 | 28.1 | - |
| Missed weeks | 14.7 | 12.6 | 1.9 weeks |
| Mean productivity costs per week | €1065 | €1065 | - |
| Productivity loss in **1.9** weeks | €2023 | €2023 | - |

In this scenario German patients work the same number of hours per week (28.1) as Dutch patients, and the mean productivity costs per hour are also the same.
